# Peripheral immune responses to filoviruses in a reservoir versus spillover hosts reveal transcriptional correlates of disease

**DOI:** 10.3389/fimmu.2023.1306501

**Published:** 2024-01-08

**Authors:** Jonathan C. Guito, Catherine E. Arnold, Amy J. Schuh, Brian R. Amman, Tara K. Sealy, Jessica R. Spengler, Jessica R. Harmon, Joann D. Coleman-McCray, Mariano Sanchez-Lockhart, Gustavo F. Palacios, Jonathan S. Towner, Joseph B. Prescott

**Affiliations:** ^1^ Viral Special Pathogens Branch, Division of High-Consequence Pathogens and Pathology, Centers for Disease Control and Prevention, Atlanta, GA, United States; ^2^ Biological Defense Research Directorate, Naval Medical Research Center, Frederick, MD, United States; ^3^ RD-CBR, Research and Development Directorate, Chemical and Biological Technologies Directorate, Research Center of Excellence, Defense Threat Reduction Agency, Fort Belvoir, VA, United States; ^4^ Center for Genome Sciences, Molecular Biology Division, U.S. Army Medical Research Institute of Infectious Diseases, Fort Detrick, MD, United States; ^5^ Department of Microbiology, Icahn School of Medicine at Mount Sinai, New York, NY, United States; ^6^ Center for Biological Threats and Special Pathogens, Robert Koch Institute, Berlin, Germany

**Keywords:** viral reservoir, spillover host, bats, Marburg virus, filovirus, immune response, comparative analysis, correlates of disease

## Abstract

Several filoviruses, including Marburg virus (MARV), cause severe disease in humans and nonhuman primates (NHPs). However, the Egyptian rousette bat (ERB, *Rousettus aegyptiacus*), the only known MARV reservoir, shows no overt illness upon natural or experimental infection, which, like other bat hosts of zoonoses, is due to well-adapted, likely species-specific immune features. Despite advances in understanding reservoir immune responses to filoviruses, ERB peripheral blood responses to MARV and how they compare to those of diseased filovirus-infected spillover hosts remain ill-defined. We thus conducted a longitudinal analysis of ERB blood gene responses during acute MARV infection. These data were then contrasted with a compilation of published primate blood response studies to elucidate gene correlates of filovirus protection versus disease. Our work expands on previous findings in MARV-infected ERBs by supporting both host resistance and disease tolerance mechanisms, offers insight into the peripheral immunocellular repertoire during infection, and provides the most direct known cross-examination between reservoir and spillover hosts of the most prevalently-regulated response genes, pathways and activities associated with differences in filovirus pathogenesis and pathogenicity.

## Introduction

Bats are unique among mammals due to a role as pollinators for some species, adaptations like powered flight and their ability to harbor and transmit a high number of viruses without clinical disease signs. As these zoonoses sometimes cause severe, life-threatening illness in humans and nonhuman primates (NHPs), specific bat reservoirs pose a major public health challenge due to the risk of spillover to vulnerable host populations (1). Infection without overt disease in bat reservoirs is likely facilitated by highly-adapted immune responses that have coevolved over millennia in a species- and virus-specific relationship, contrasting with the dysfunctional responses of spillover hosts that lack the adaptations needed to limit replication by zoonotic viruses while protecting against immunopathology (1–9). A prime example of host pathogenicity differences are filoviruses like Marburg virus (MARV), for which the Egyptian rousette bat (ERB, *Rousettus aegyptiacus*) is the only verified filovirus reservoir, and which, along with other filoviruses like Ebola virus (EBOV) and Sudan virus (SUDV), cause sporadic, deadly outbreaks in primate spillover hosts (7, 10–14).

Filovirus disease in humans and NHPs involves responses by immune cells early in infection that are important for viral control and clearance as well as for viral pathogenesis (7, 15–19). Initial targeted infection and dysregulation of macrophages and dendritic cells (DCs) in the blood permits uncontrolled filovirus replication in tissues and drives runaway pro-inflammatory response activation (cytokine storm), contributing to tissue pathology followed by often-fatal disease manifestations (e.g., vascular leakage and disseminated intravascular coagulation [DIC]) (7, 15–20). Conversely, ERBs quickly control MARV replication, mostly clearing virus within two weeks post-infection and exhibiting no obvious clinical signs of inflammation or pathology (21–24). Multiple gene expression analyses of ERB immune responses to MARV infection suggest roles for host resistance mechanisms to control replication and prevent virus-mediated disease, and for disease tolerance mechanisms to control inflammation and prevent host immune-dysregulated disease (6, 25–28). However, no analysis of MARV-infected ERBs has yet investigated blood immune responses, which we hypothesized would show differentially expressed genes (DEGs) related to both mechanisms, including DEGs previously observed in bat tissues (e.g., liver) and those unique to blood’s diverse immunocellular milieu. Furthermore, a powerful benefit of quantitating reservoir responses to coevolved virus infection is the ability to discern how they contrast with the maladapted responses of spillover hosts, which is especially important given that *in vivo* bat response studies have typically been standalone and descriptive, with a lack of disease correlative context that makes specific immunological conclusions difficult (6, 23, 29–36). Such comparisons also deepen understanding of primate infection by absolving any gene responses shared with overt disease-free reservoirs as unlikely to be virulence determinants. Unfortunately, *in vivo* elucidation of shared and divergent filoviral responses between reservoirs and disease hosts has been complicated by a lack of bat research tools and comparable datasets. Blood-based transcriptional analysis historically has been the most utilized approach for measuring primate responses, the only biologically reliable way available to retrospectively assess host responses across studies (10, 37–45). However, comparative analyses even among multiple existing primate datasets have been surprisingly limited in the literature (40, 44–46), and would likewise help highlight the top commonly dysregulated genes and pathways most deserving of further scrutiny.

Given the considerable knowledge gaps, it was prudent to identify immune-related genes that were consistently dysregulated in filovirus spillover hosts and which of these DEGs were shared by, or divergent from, a natural bat reservoir. Therefore, using an array of ERB-specific gene probes following MARV inoculation of naïve bats, we temporally evaluated peripheral transcriptional host responses and compared them side-by-side to cognate responses previously reported for filovirus-infected primates. Additionally, while other *in vivo* ERB studies have been serial euthanasia experiments, necessitating destructive sampling of time point-based bat cohorts (6, 22, 23, 30, 31), this work allowed us to quantify responses within the same individual bats nondestructively, mitigating potential cohort-to-cohort variation and better resolving within-host response progression. Our findings highlight the exquisite control, sensitivity and specificity of blood immune responses in the MARV bat host and offer the most direct *in vivo* response comparison available between and among filovirus-infected reservoir and spillover hosts. Together, they provide a more comprehensive picture of the most commonly-regulated genes and immunological dynamics correlated with filoviral pathogenesis and pathology.

## Methods

### Biosafety and animal care

Work with ERBs and MARV was conducted by highly trained laboratorians under Biosafety Level 4 (BSL4) high containment at CDC in accordance with animal and select agent regulations, protocols and practices, overseen by CDC’s Institutional Animal Care & Use Committee (IACUC), Animal Care and Use Program Office (ACUPO) and Comparative Medicine Branch (CMB). Bats were housed in primate-sized caging within HEPA-filtrated Duo-Flow mobile bioisolator units (Lab Products, Inc.) and given food and water *ad libitum* with 12-hour day/night light cycles. Bats were allowed to acclimate to the BSL4 environment for at least five days prior to experimental manipulations. Animal husbandry was performed daily as previously described (6, 23, 31).

### MARV inoculation, bat sampling and virus quantitation

Prior to infection, to establish baseline control values of bat gene expression, we collected whole blood from the cephalic vein on the wings of five naïve juvenile (~8-9-month-old) outbred, captive-bred ERBs previously moved into the BSL4 animal suite. These bats were then inoculated subcutaneously under isoflurane inhalation anesthesia with 10^4^ TCID_50_ (50% tissue culture infectious dose) of ERB viral isolate MARV-371bat as previously described (6, 21, 23). Whole blood samples were collected daily from alternate wings of each of the five bats between 1 day post-infection (DPI) and 14DPI, as well as two weeks later at 28DPI. Blood samples were immediately placed into MagMAX lysis buffer (ThermoFisher Scientific) for virus inactivation and safe removal from BSL4 containment. Total RNA was extracted using the MagMAX-96 Total RNA Isolation kit and a MagMAX Express-96 processor. We performed quantitative reverse-transcription PCR (qRT-PCR) using MARV *VP40* primers and probes, positive and negative technical controls and an *18S* rRNA reference gene control. TCID_50_ equivalent values per mL were based on extrapolation of standard curves generated from serial dilution of a known amount of MARV. The remaining RNA was stored at -80°C.

### Whole blood RNA processing

Total RNA from each sample was thawed and added to NanoString nCounter reagents, including Reaction CodeSet and Capture ProbeSet, which together feature our custom array of 380 ERB immune-related gene probes (CodeSet) (6). Multiple sets of 12 reaction mixes were hybridized in PCR tubes on a thermal cycler (Bio-Rad) at 65°C for 24 hours, then cooled to 4°C. Each set was removed from the thermal cycler, nuclease-free ddH20 was added to reaction mixes, and mixed samples were pipetted into a NanoString cartridge. Each cartridge was loaded onto an nCounter SPRINT machine and run according to manufacturer’s instructions.

### Whole blood transcriptional analysis using ERB immune-related gene CodeSet

Following each nCounter run, blood sample data files containing raw mRNA counts for ERB CodeSet gene targets were uploaded into the nSolver 4.0 software system and initially processed for quality. Out of a total of 80 bat whole blood samples across the longitudinal infection (five bats x 15 time points post-infection, plus the five pre-bleed control samples), 77 samples passed QC; three samples (one each at 1DPI, 14DPI and 28DPI) were excluded from further analysis due to low RNA yield and insufficiently robust nCounter binding densities. The remaining samples were processed for positive and negative technical control normalization before differential gene expression quantitation with nCounter Advanced Analysis Module 2.0. This calculated fold-changes (FC) for DEGs averaged across bats at each time point using the “Optimal” setting and was normalized based on nSolver’s determination of the five most suitable (least variable) reference genes via geNorm (*RIPK1*, *SUMO1*, *PTPN2*, *HPRT1* and *RAB5A*). DEGs were further vetted for above-background expression (normalized counts > 24), biological relevance (FC ≥ ±2.0) and significance (adjusted p-value ≤ 0.05) as previously described (6, 28, 47). Six genes with ubiquitous borderline or below-background count threshold levels were excluded from analysis as previously described: *IFI44*, *IFITM2*, *IL12A*, *IL21*, *IL3* and *TLR9 (* 6, 28, 47).

### Heatmap generation, pathway analysis and immune cell type profiling

A heatmap of significant, relevant and above-background DEGs of the bat blood response time course was generated using the Morpheus software as previously described (6, 28, 47), with a downregulated (blue) FC range from -10 (0.1) to -2 (0.5) and upregulated (red) FC range from 2 to 100. Only datapoints meeting all three criteria for any DEG at any given time point were plotted on the heatmap. For determining significantly enriched pathways, the Reactome database was utilized (https://www.reactome.org) (48), and only pathways containing at least three DEGs and an Entities FDR (false discovery rate) adjusted p-value ≤ 0.05 were considered. Pathways unrelated to filovirus infection specifically or generally (e.g., oncological, specific to other infections/diseases) were removed from further analysis. Figures for pathway analysis were generated using the ggplot2 package (version 3.4.2) in R (version 4.2.1). CIBERSORTx web-based software analysis (49) (https://cibersort.stanford.edu/, Stanford University) to estimate changes in immune cell populations over time was based on the input of the average linear gene expression counts at each time point for all normalized immune-related genes on the ERB CodeSet and was performed as previously described (6). For this study, 500 permutations were performed, and an existing mouse signature matrix was used (50).

### Cross-species comparative analysis

Nine published NanoString (NS), microarray (MA) or RNA-sequencing (Seq) based datasets from eight studies containing FC and significance values for NHP/human whole blood or peripheral blood mononuclear cell (PBMC) gene responses were obtained from online databases, supplemental data files or individual corresponding authors: Woolsey 2022 (51) (MARV-Angola, NS, intramuscularly [IM] inoculated control NHPs only); Marzi 2019 (40) (MARV-Angola, Seq, IM inoculated VSV-EBOV-vaccinated control NHPs only); Connor 2015 (52) (MARV-Angola, PBMCs, MA, aerosol inoculated); Woolsey 2022 (53) (SUDV-Gulu, NS, IM inoculated); Rubins 2007 (43) (EBOV-Kikwit, PBMCs, MA, IM inoculated); Speranza 2017 and 2018 (44, 54) (EBOV-Makona, Seq and NS, IM and intranasally [IN] inoculated [normal IN infection profile NHPs only]); and Liu 2017 (39) (EBOV-Makona, Seq). These transcriptional datasets were originally produced using samples collected from experimental infection of cynomolgus and/or rhesus macaques, or for Liu 2017, using anonymized samples collected from over 100 acutely EBOV-infected patients by the European Mobile Laboratory in Guinea in 2014-2015 and matched by similar Ct values that alone were not predictive of clinical outcome. For microarray datasets, reanalysis of FC and significance was performed using the Gene Expression Omnibus (GEO) database (GSE58287 [Connor 2015] and GSE8317 [Rubins 2007]) at each time point post-infection in comparison to control samples, each producing a separate downloadable data file. For datasets containing FC and adjusted p-values, log_2_ FC was converted to linear FC where applicable, and datasets were refined to include only the matching 380 host gene targets present on the ERB CodeSet to make cross-comparison possible. Some gene targets were not identifiable by common gene name within a given dataset; multiple alternative gene names were attempted before ruling out their presence in that dataset and excluding them from further analysis (represented by asterisks in heatmaps). As only significant and relevant DEGs were present in the dataset from Liu 2017, exclusion of non-identifiable genes was not performed and asterisks were not included, since it cannot be discerned if lack of fold-change for any such gene indicates absence of up- or downregulation or absence of any measurement. For microarray data, if multiple instances of the same gene were identified, their significant FC values were averaged. Dataset spreadsheets were then processed similarly to the ERB nCounter data, with DEGs identified based on the same biological relevance and significance criteria as described above for ERBs for each time point or condition. For all significant DEG data, FC values were compiled across datasets and heatmaps were generated as described above, with an upregulated FC range from 2 to 250.

### Statistics

FC values of significant DEGs following nCounter Advanced Analysis or comparative analysis were based upon Benjamini-Yekutieli (ERB and published microarray datasets) or Benjamini-Hochberg (FDR) adjusted p-values of ≤ 0.05; Reactome analysis of significant pathways was based upon an Entities FDR adjusted p-value of ≤ 0.05.

## Results

Blood transcriptional immune responses to filoviruses such as MARV, EBOV and SUDV have been repeatedly studied for vulnerable spillover hosts like humans and NHPs. However, for ERBs, the MARV reservoir, such peripheral immune responses are currently uncharacterized. To investigate these responses, we inoculated five naïve juvenile captive-bred ERBs with MARV and collected wing whole blood samples temporally for virus quantitation and immune-related differential gene expression analysis. Since sampling was nondestructive, we utilized the same individual bats for the entirety of our analysis.

**Figure 1:**
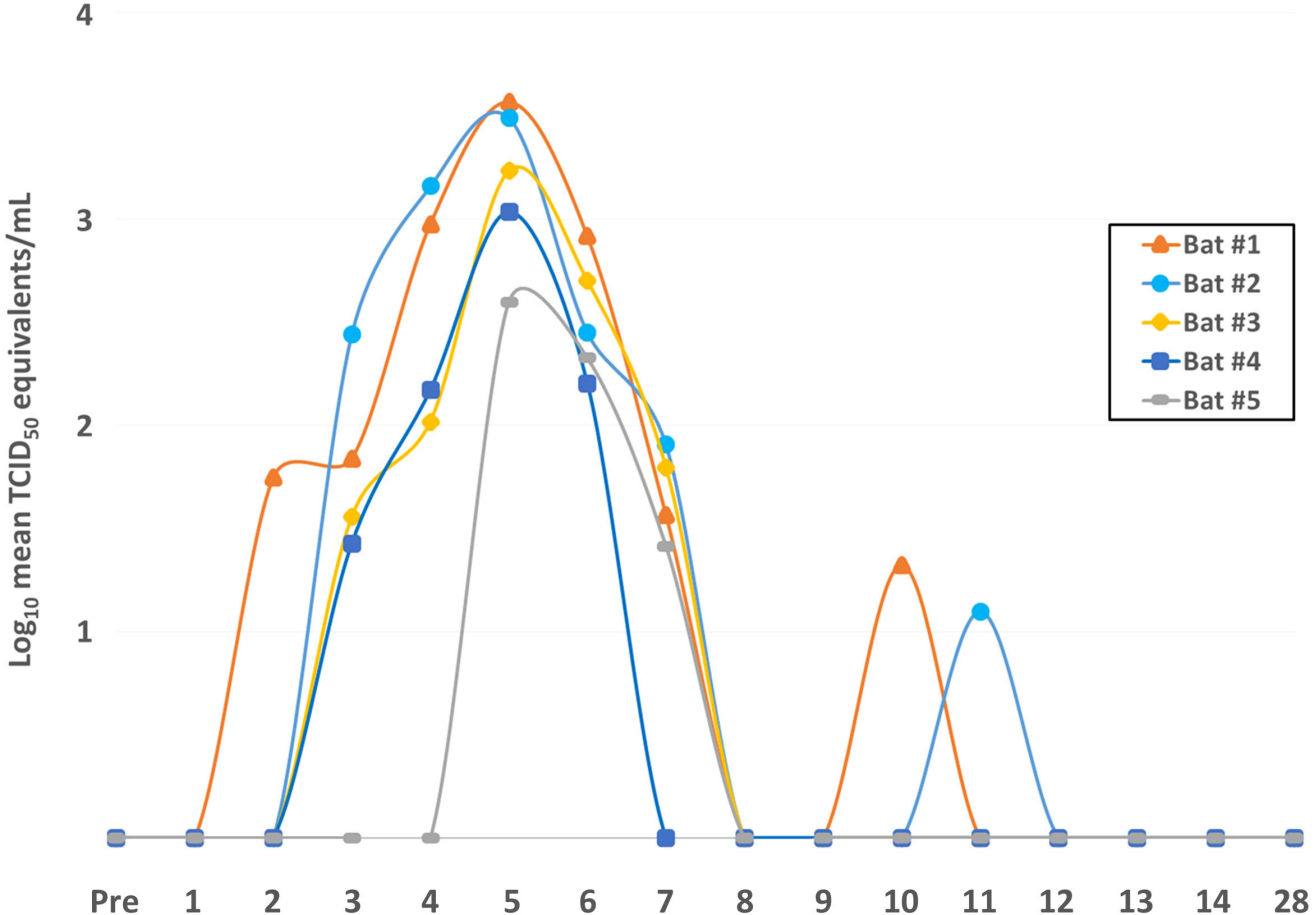
Daily MARV loads in the blood of individual ERBs. Blood from the wings of each of the five bats in the study was collected daily before (Pre = pre-bleed) and after MARV inoculation through 14DPI and again at 28DPI, total RNA was extracted, and quantitative reverse-transcription PCR was performed using MARV-specific primers and probes. Ct values were converted to geometric mean log_10_ TCID_50_ eq./mL using a standard curve of a known quantity of virus. MARV loads in the blood are shown for each bat over time as indicated by line color and data point symbol.

Consistent with prior ERB infection studies (6, 21, 23), the bats harbored robust MARV RNA loads in the blood during the acute phase of infection, with initial MARV detection by qRT-PCR at 2DPI that increased daily to peak viral loads in all bats by 5DPI (over 3 logs geometric mean TCID_50_ equivalents/mL) (**Figure 1**). This was followed by a rapid decrease of MARV loads until qRT-PCR-negativity as early as 8DPI. MARV became consistently undetectable in all bats by 12DPI. On 10DPI and 11DPI, one animal for each time point, Bats #1 and #2, respectively, harbored low MARV loads. Bats #1 and #2 had the strongest overall viral RNA loads in the study, up to 3.7x10^3^ and 3.1x10^3^ TCID_50_ eq./mL, respectively, with a peak MARV load at 5DPI, and Bat #1 was the earliest bat to show qRT-PCR-detectable virus levels, at 2DPI. Conversely, Bats #4 and #5 had the lowest peak MARV loads, at 1x10^3^ and 4x10^2^ TCID_50_ eq./mL, respectively, at 5DPI.

**Figure 2:**
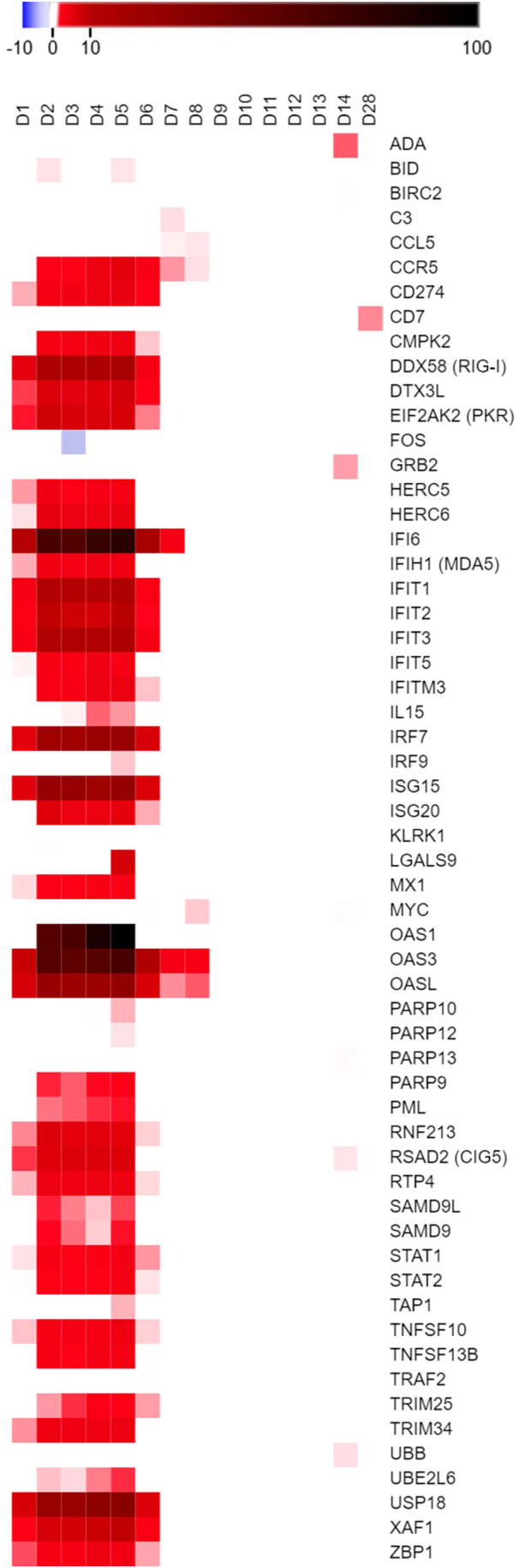
ERB blood transcriptional immune response to MARV longitudinally among the same bats. Heatmap of DEGs from the five MARV-infected ERBs in the study shown as linear fold-change (FC) averaged across bats at each time point in comparison to naïve control blood samples (pre-bleeds), with only DEGs from the ERB nCounter CodeSet that were identified as both relevant (FC ≥ ±2) and significant (adjusted p-value ≤ 0.05) at any given data point plotted on the heatmap (red = upregulation, blue = downregulation) as further detailed in Methods. FC scale bar = -10 to -2, 2 to 100.

After confirming that MARV-infected ERBs possessed MARV RNA loads in the blood, we used the nCounter platform with a CodeSet of 380 ERB-specific immune-related gene probes to investigate peripheral transcriptional responses to MARV. Our analysis ultimately identified 58 differentially expressed genes (DEGs) in whole blood across the ERB infection time course that met significance (adjusted p-value ≤ 0.05), biological relevance (linear fold-change [FC] ≥ ±2) and background count (> 24) criteria (**Figure 2**, **Supplementary Data File 1**). Significant peripheral responses consisted almost exclusively of gene upregulation, with only two DEGs, *KLRK1* and *FOS*, being minimally downregulated (2- to 2.5-fold) at 2DPI and 3DPI, respectively. Gene induction for nearly half the DEGs began as early as 1DPI, preceding initial MARV RNA detection, and rapidly rose to a relatively stable level of activation for the majority of DEGs by 2DPI. Regulation was maintained through peak infection at 5DPI, then activation levels rapidly declined for most DEGs, particularly for those that were not significantly or only modestly activated at 1DPI. Of the 26 genes showing sustained upregulation through 6DPI, 21 were initially activated at 1DPI. By 7DPI, four of these genes remained significantly induced, and by 8DPI, only three remained, *CCR5*, *OAS3* and *OASL*, with DEGs returning to nonsignificant and/or baseline levels beyond 8DPI save for modest, sporadic activation of a handful of genes.

*OAS3* and *OASL* showed the most sustained activation in MARV-infected ERB blood, with elevated levels throughout the first eight days; *IFI6* was the second-most sustained, upregulated until 7DPI. Another OAS family member, *OAS1*, was the highest-activated DEG in the study, its levels peaking at 4-5DPI. *IFI6* was the second-highest induced gene, followed by *OAS3* and, at considerably lower intensities, by *ISG15*, *USP18*, *OASL*, *IRF7* and *DDX58* (*RIG-I*). Other notable genes with sustained, low-to-moderate activation included *CD274*, *EIF2AK2* (*PKR*), *HERC5*/*6*, *IFIH1* (*MDA5*), various *IFIT*s, *MX1*, *PARP9*, *SAMD9*/*9L*, *STAT1*/*2*, *TNFSF10*/*13B*, *TRIM25*/*34*, *UBE2L6*, *XAF1* and *ZBP1*. Several genes also showed sporadic, if mainly low-level, upregulation, including *BID* at 2DPI and 5DPI, *IL15* from 3-5DPI, *LGALS9* at 5DPI, *ADA* and *GRB2* at 14DPI and *CD7* at 28DPI (the only activated DEG at endpoint). Immune responses were generally consistent across individual bats, although some time- and gene-dependent expression trends were noted for specific bats that were either correlative to, or distinct from, their recorded viral loads (**Supplementary Figure 1**).

**Figure 3:**
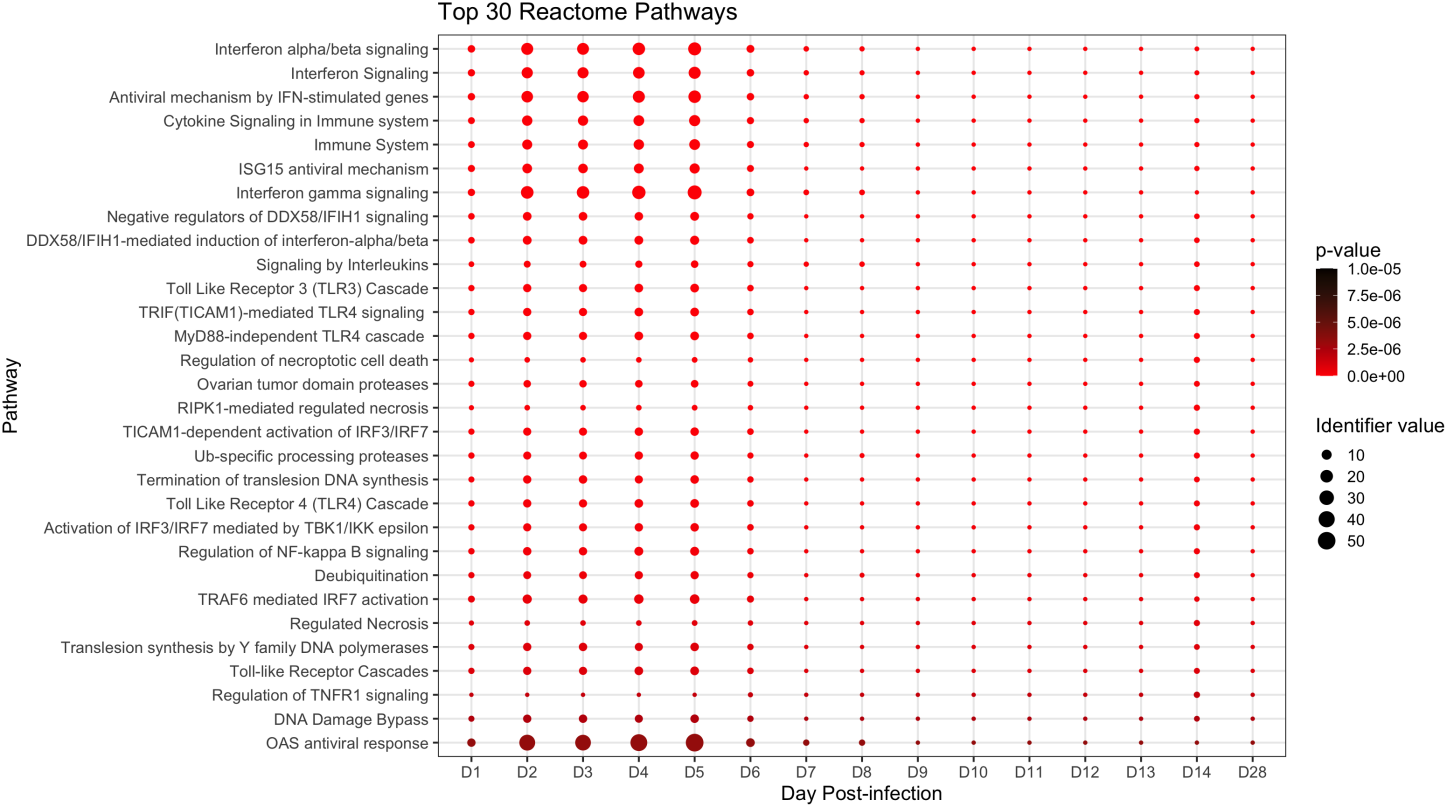
Top significant pathways up- or downregulated during the ERB peripheral response to MARV. Pathway analysis via the Reactome database with enriched pathways plotted by day post-infection. All above pathways are constituted by at least three DEGs and have an overall adjusted p-value ≤ 0.05 as described in Methods, with p-value color shifting to lighter red as significance increases. Identifier value = overall pathway-level FC. Image generated using the ggplot2 package in R software. Full analysis of all 78 significantly enriched pathways can be found in **Supplementary Data File 2**.

We next conducted pathway analysis using Reactome to obtain a broader, higher-level view of peripheral immune responses in ERBs. Reactome identified 78 significant pathways, the top 30 of which are presented in **Figure 3** (full analysis in **Supplementary Data File 2**). Among top scorers were antiviral interferon (IFN) signaling gene (ISG) pathways (especially OAS response (27, 55)), cytokine/pro-inflammatory signaling pathways, and other innate and adaptive immune-related pathways. Additional relevant pathways during MARV infection included signaling by G-CSF, by TNF, by M-CSF in myeloid cells and by TGFβ family members; the TNFR2 non-canonical NFκB pathway; multiple antigen processing-, DNA damage/repair- and cell death-related pathways; and signaling of interleukins (including IL1, 2, 4 and 13), TLRs (including TLR1, 2, 5, 6 and 10) and MAPKs (**Supplementary Data File 2**).

**Figure 4:**
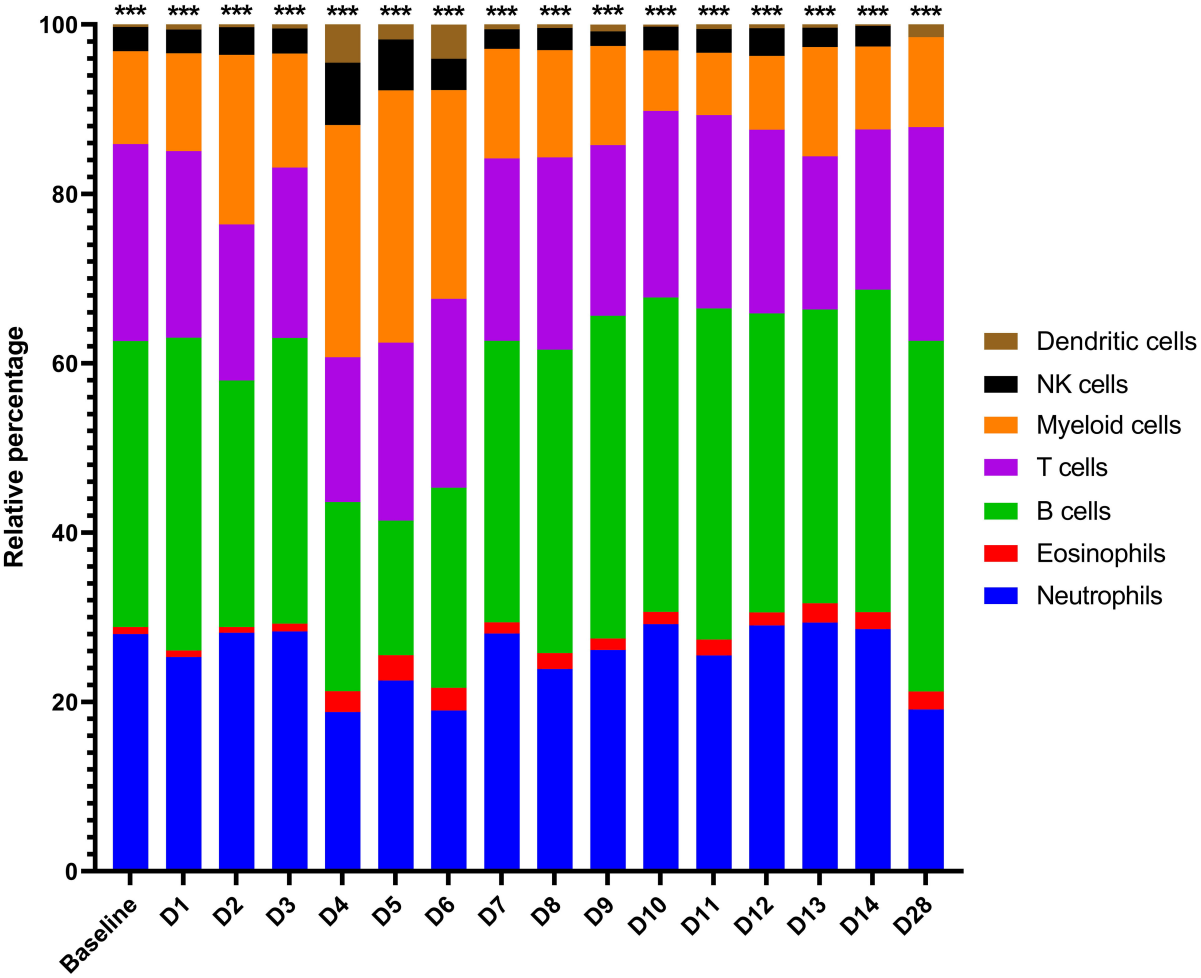
CIBERSORTx gene expression-based proportional immunocellular profiling during ERB peripheral response to MARV. CIBERSORTx web-based software analysis was used to estimate proportional changes in immune cell populations over time prior to and each day post-MARV infection. Analysis was based on the input of average linear gene expression counts at each time point for all normalized immune genes on the ERB nCounter CodeSet as described in Methods. For this study, an existing mouse signature matrix was used to map expression of immune gene markers to particular immune cell types/lineages, as indicated by color. Asterisks at top are an output of CIBERSORTx denoting the level of confidence in the algorithm having correctly deconvoluted the cellular populations (< 0.0005).

Given that the blood acts as a conduit for various critical immune cell types, including after pathogen-mediated activation and proliferation (e.g., monocytes/macrophages, DCs, neutrophils and T, B and natural killer [NK] lymphocytes), we sought to assess how these cell populations changed proportionally in MARV-infected bats over time (3, 11, 16, 43, 44, 52, 56). This was accomplished by transcriptomic cell type profiling using CIBERSORTx with normalized ERB gene expression data, which along with hematology-based analysis in whole blood (23), has been previously performed to analyze immune cell populations for MARV-infected ERBs in the skin and spleen (6). Here, in naïve bat whole blood, baseline proportions were estimated by CIBERSORTx for several peripheral immune cell types or lineages, including for myeloid cells (monocytes/macrophages, ~11%), DCs (<1%), NK cells (~3%), T cells (~23%), B cells (~33%), eosinophils (~1%) and neutrophils (~28%) (**Figure 4**), consistent with published single-cell RNA sequencing analysis of naïve ERB blood (57). Upon MARV infection, there was a shift in cellular proportions, particularly during peak viral loads (4-6DPI), involving expansions of myeloid cells, DCs, NKs and eosinophils, a slight decrease in T cell and neutrophil proportions, and a substantial reduction in B cell proportions. As MARV replication was controlled past 6DPI, most cellular proportions, with minor discrepancies (e.g., DCs and neutrophils at 28DPI), returned to baseline or near-baseline levels, correlating with resolution of acute phase infection and viral clearance.

**Figure 5:**
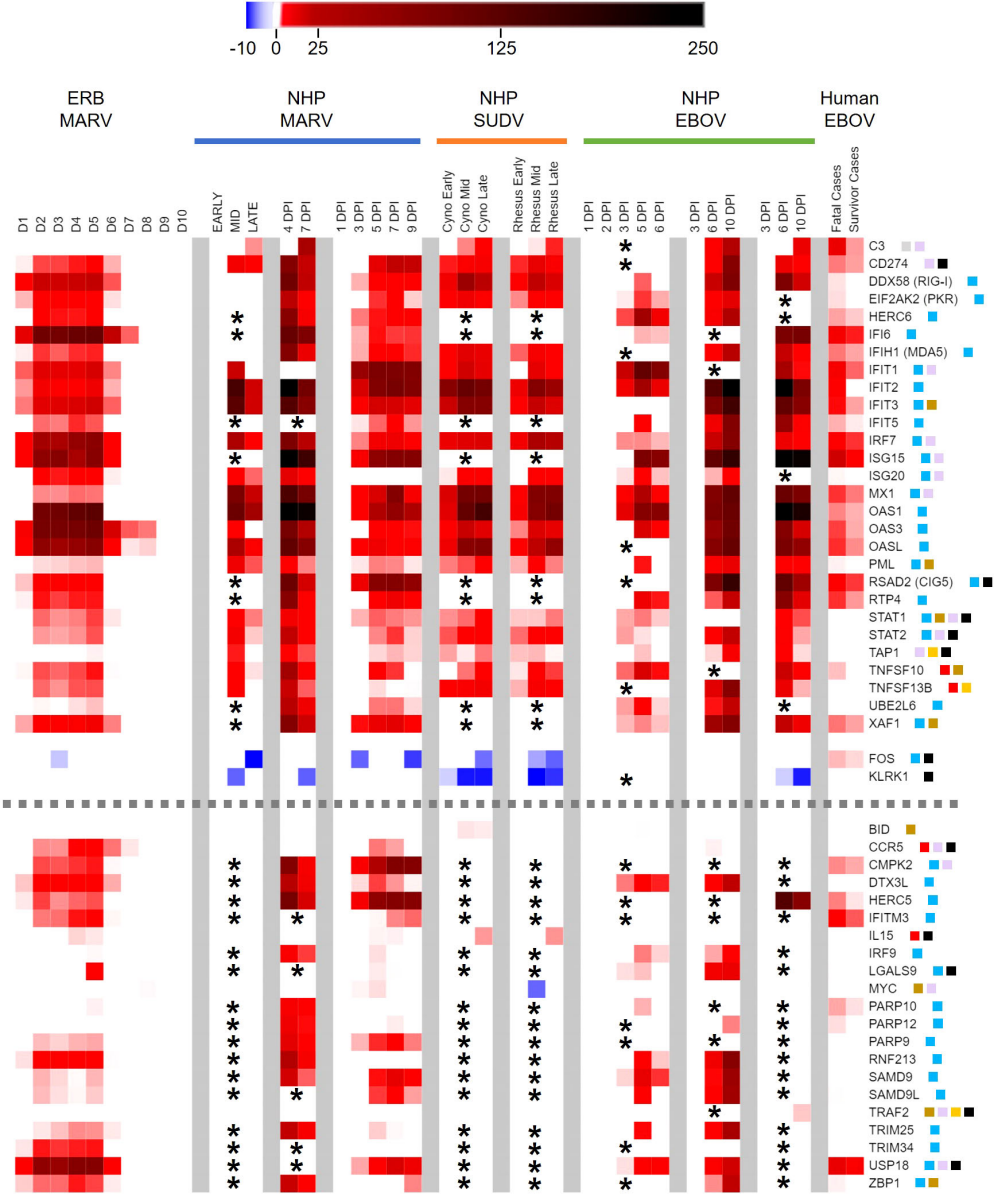
Peripheral immune-related gene responses to filoviruses shared among reservoir and spillover hosts. Heatmap as in **Figure 2** of comparison of ERB blood response DEGs to DEGs from published whole blood/PBMC datasets of NHP (cynomolgus or rhesus macaques) or human responses. Nine primate datasets from eight studies are arranged from left to right as follows (separated by grey column breaks): Woolsey 2022 (MARV-Angola); Marzi 2019 (MARV-Angola); Connor 2015 (MARV-Angola); Woolsey 2022 (SUDV-Gulu, Cyno and Rhesus); Rubins 2007 (EBOV-Kikwit); Speranza 2017 and 2018 (EBOV-Makona); and Liu 2017 (EBOV-Makona); these datasets are described in more detail in Methods. For the MARV Woolsey study, Early = 3DPI, Mid = 6DPI and Late = 10DPI; for the Woolsey SUDV study, Early = 3-4DPI, Mid = 5-6DPI and Late = 7-10DPI; for the Liu EBOV study, human patient samples were collected during the acute phase of illness. All datasets are shown as averaged linear FC and consider only the 380 response gene targets present on the ERB CodeSet. Only DEGs that were identified as both relevant (FC ≥ ±2) and significant (adjusted p-value ≤ 0.05) at any given data point were plotted. Some gene targets were not identifiable within a given dataset and were excluded from analysis (represented by asterisks). For Liu 2017, exclusion of non-identifiable genes could not be determined from the dataset information and asterisks were not included; therefore, lack of plotted FC for genes in this dataset indicates neither absence of up- or downregulation nor absence of overall measurement. Dotted grey lines denote separation between tiers of DEG consistency across datasets: the upper tier are those shared DEGs that were identified across at least five datasets, while the bottom tier are those shared DEGs that were identified with less consistency across datasets. FC scale bar = -10 to -2, 2 to 250. Color-coded boxes represent functional associations of DEGs related to responses for: antiviral/IFN/ISG (blue); pro-inflammatory cytokine/regulator/cell marker (red); anti-inflammatory cytokine/regulator/cell marker (green); cell survival/trafficking/stress (brown); neutrophil (grey); monocyte/macrophage/DC (purple); B cell (orange); T/NK cell (black).

**Figure 6:**
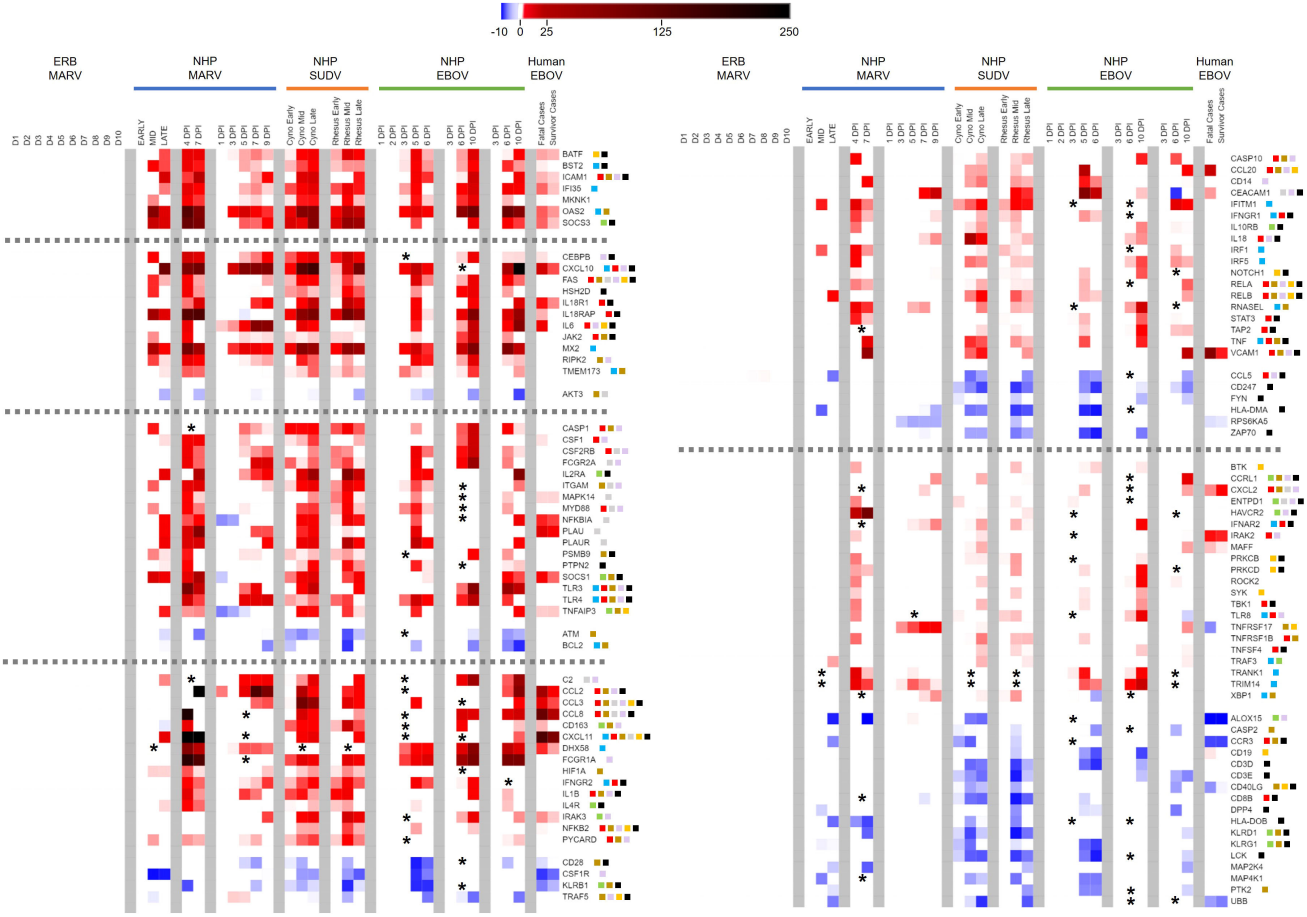
Peripheral immune-related gene responses to filoviruses divergent between reservoir and spillover hosts. Heatmap as in **Figures 2**, **5** of ERB blood response DEGs compared to DEGs from published datasets of NHP/human blood responses as described in **Figure 5** and Methods. All datasets are shown as averaged linear FC and consider only the 380 gene targets on the ERB CodeSet. Only DEGs that were identified as both relevant (FC ≥ ±2) and significant (adjusted p-value ≤ 0.05) at any given data point were plotted. Some genes were not identifiable in a given dataset and excluded from analysis (represented by asterisks). For Liu 2017, exclusion of non-identifiable genes could not be determined from the dataset information and asterisks were not included; therefore, lack of plotted FC for genes in this dataset indicates neither absence of up- or downregulation nor absence of overall measurement. Dotted grey lines denote separation between tiers of DEG consistency across primate datasets, from (left-side heatmap) all nine datasets at top to six at bottom, and from (right-side heatmap) five datasets at top to four at bottom. FC scale bar = -10 to -2, 2 to 250. Color-coded boxes represent functional associations of DEGs related to responses for: antiviral/IFN/ISG (blue); pro-inflammatory cytokine/regulator/cell marker (red); anti-inflammatory cytokine/regulator/cell marker (green); cell survival/trafficking/stress (brown); neutrophil (grey); monocyte/macrophage/DC (purple); B cell (orange); T/NK cell (black). Full comparative DEG analysis can be found in **Supplementary Data File 1**.

Since no side-by-side assessment of immune responses with comparable datasets has yet been performed between a filovirus reservoir and spillover hosts, we took advantage of our ERB blood transcriptional dataset and compared it to a compilation of published primate blood response datasets (39, 40, 43, 44, 51–54). Evaluating nine datasets from eight studies of MARV-, EBOV- or SUDV-infected cynomolgus or rhesus macaques or EBOV-infected human patients, we first examined only the 380 immune-related gene targets present on our ERB CodeSet, then assessed DEGs (up to 10DPI for all but the human dataset) based on the bat FC and significance criteria. Resultant comparative heatmaps show either significant filoviral immune responses shared across datasets regardless of host (**Figure 5**) or shared between primates but exclusive of ERBs (**Figure 6**), stratified in each heatmap by the prevalence of each DEG regulated across a descending number of datasets.

Examining the common gene responses between hosts (**Figure 5**), MARV-infected ERBs shared nearly all their identified DEGs with multiple filovirus-infected primate datasets. The majority of DEGs common between primates and ERBs (n=30) were identified in at least five of the primate datasets with available FC information and in some cases in every dataset examined (**Figure 5**, top tier), with similar magnitudes and kinetics of up- or downregulation among primate datasets despite variable study designs. The inconsistent regulation of 16 of the 21 other DEGs shared between ERBs and only four or fewer primate datasets (**Figure 5**, bottom tier) was due to a lack of obtainable expression values in certain datasets for those genes. Despite this data gap, overall results were robust enough for evaluation.

Strikingly, many ERB DEGs (e.g., *RIG-I*, *PKR*, *OAS1*), regardless of frequency across datasets, were also induced to similar magnitudes as for primates. However, DEG induction in ERBs occurred earlier and was more transient, returning to baseline levels by 7DPI except for very few early- and highly induced DEGs (*IFI6*, *OAS3*, *OASL* and *CCR5*), by which point disease in primates becomes severe. Of the five inconsistently-regulated ERB DEGs (**Figure 5**, bottom tier) based on a true lack of significant induction (i.e., had obtainable expression values) across most primate studies, a notable deviation in expression between hosts was *CCR5*, which was activated in ERBs at higher and more sustained induction levels than in primates. *USP18* was the only other DEG that showed sustained stronger expression in ERBs, with at least double the level of induction as that seen in primates. Otherwise, expression difference trends between hosts were due to ERBs having overall lower levels of regulation (e.g., *C3*, *IFIT2*, *MX1*, *PML*, *UBE2L6*, *FOS*, *KLRK1*, *PARP9*, *SAMD9*, *SAMD9L*). For the primate responses shared with ERBs, some DEGs showed mainly sustained or stronger activation (e.g., *CD274*, *IFI6*, *ISG15*, *MX1*, *OAS1*) when transitioning from mid- to late-stage infection, while other DEGs showed mainly reduced activation (e.g., *IFIT1*, *PML*, *TAP1*), but for most of these shared primate DEGs, the level of activation between disease stages was study-dependent. Finally, we observed similar or slightly weaker responses for fatal human cases compared to those from NHPs and ERBs, with divergent upregulation in humans for *FOS*. Meanwhile, human survivors showed reduced or absent differential expression for additional genes identified for NHPs and ERBs, including *RIG-I*, *IFIT2*, *ISG20*, *PML*, *TNFSF10*, *LGALS9*, *SAMD9L* and *ZBP1*.

Next, we examined DEGs that diverged in expression between reservoir and spillover hosts. We identified nearly 300 out of the 380 ERB CodeSet-matched gene targets as significant primate-specific DEGs in at least one of the nine primate datasets (**Supplementary Data File 1**). Of these, 119 DEGs were present across at least four datasets, again with marked overall consistency in their magnitudes and kinetics (**Figure 6**). Indeed, only 12 out of 3,451 individual data points, for 12 different DEGs from one dataset each, dissented from the up- or downregulation trend observed for that DEG across all other datasets for which it was significantly expressed (e.g., *IL18RAP*, *NFKBIA*, *CD163*, *TRAF5*, *CEACAM1*). In general, upregulation was much more common than downregulation, especially as DEGs became more prevalent across a greater number of datasets. Seven DEGs were upregulated across all nine primate datasets: *BATF*, *BST2*, *ICAM1*, *IFI35*, *MKNK1*, *OAS2* and *SOCS3*. These were followed by DEGs regulated in seven or eight datasets, both upregulated (e.g., *CEBPB*, *FAS*, *IL18R1*, *IL6*, *MX2*, *CASP1*, *CSF1*, *IL2RA*, *MYD88*, *SOCS1*, *TLR3*/*4*) and downregulated (*AKT3*, *ATM* and *BCL2*). Several DEGs were less consistent and regulated in fewer datasets, either upregulated (e.g., *CCL2*/*3*/*8*, *CD163*, *FCGR1A*, *IL1B*, *IL4R*, *NFKB2*, *CD14*, *IL10RB*, *RNASEL*, *TNF*, *VCAM1*, *CXCL2*, *HAVCR2*, *RELA*/*B*, *TRANK1*) or downregulated (e.g., *CD28*, *CSFR1*, various *KLR*s, *CCL5*, *HLA*-*DMA*/*DOB*, *CD19*, *CD3D*/*E*, *CD8B*, *CD40LG*). Of all DEGs, *CCL5* (*RANTES*) was the only significant ERB gene that completely diverged in FC from the spillover hosts, showing modest upregulation for ERBs but consistent downregulation among primate datasets. For the human dataset, survivors showed several DEGs with reduced or absent differential expression compared to fatal cases, more closely resembling the bat reservoir. These DEGs included *BST2*, *SOCS3*, *CEBPB*, *IL18R1*, *IL18RAP*, *IL6*, *CSF1*, *CD163*, *DHX58*, *IRAK3*, *CD28*, *CCL20* and *CEACAM1*.

Finally, to ensure the robustness of our ERB dataset, and that different significance algorithms used in some primate datasets weren’t biasing DEG elucidation, we reanalyzed ERB gene expression up to 10DPI using Benjamini-Hochberg (false discovery rate, FDR) significance. We obtained a near-identical DEG profile (**Supplementary Data File 1**). The only differences were that *CXCL10* (*IP-10*) became significantly upregulated at 5DPI, though to a lesser extent (~tenfold) than measured for primates, and that *S100A12* became significantly downregulated at 4DPI, diverging from a few primate datasets that instead showed inconsistent moderate induction. Overall, this emphasizes the high quality and reliability of our ERB blood response analysis and comparisons between ERB and primate datasets.

## Discussion

Revealing the mechanisms by which bat reservoirs actively control virus replication and protect themselves from disease, while spillover hosts cannot, has been confounded by a paucity of bat-specific research tools that would permit comparative studies (e.g., animal colonies, transcriptomes and genomes, antibodies for flow cytometry and immunohistochemistry, probes for gene expression analysis, etc.) (2, 6, 8). Consequently, attempts to compare *in vivo* immune responses have been at best indirect due to experimental design incompatibilities; limited bat accessibility, sample sizes or gene coverage; and mismatched specimen types, time points, analysis methods or analytes (e.g., protein versus gene expression) (6, 22, 23, 30, 35, 58). Conversely, most primate response studies have measured peripheral transcriptional responses. However, these analyses have been confined almost exclusively to each study’s contexts, with few reports having leveraged the availability of other datasets for compilation and comparison to better ascertain consistencies and potential distinctions between study conditions (e.g., virus isolate, primate species, etc.) (40, 44–46). Fortunately, recent efforts to establish specific tools for ERBs have greatly progressed, maturing enough to allow for our *in vivo* examination of cross-host responses presented herein (6, 23, 25, 28, 31, 47, 58–60).

This comparative blood response analysis between bat reservoir and primate spillover hosts revealed a remarkable consistency across primate studies despite notable experimental variation, including a relatively narrow yet comprehensive selection of ERB-based gene targets and differing primate species (cynomolgus or rhesus macaques or humans), inoculation routes/dosages, filovirus isolates/species (e.g., MARV-Angola, EBOV, SUDV), specimen types (whole blood, PBMCs), infection time courses and transcriptional profiling platforms (nCounter, RNA-seq, microarray). This greatly bolsters confidence in the biological accuracy of these studies in having defined the *bona fide* transcriptional mechanisms underlying filovirus infection in primates, and is a testament to the quality, precision and care taken by the investigators performing primate studies, to the benefit of the understanding of these filoviral diseases. Our comparative analysis also underscores that, although each study begets a unique overall response profile, there is nevertheless a core set of genes intrinsic to infection and pathogenesis by filoviruses, for either primates alone or for primates and ERBs. This consistency makes it possible to discern discrete mechanisms across datasets, with important implications for specific cellular immune responses, their relevance to pathogenesis among spillover hosts, and their putative contributions to disparate disease outcomes compared to those in a natural host.

Peripheral responses shared between filovirus-infected ERBs and primates were typical of mammalian viral infections and encompassed almost all the DEGs we identified for ERBs, showing strikingly similar induction of mainly canonical antiviral ISGs (e.g., *ISG15*, *MX1*, *OAS1*/*3*, *IFIT*s, *STAT1*, *FOS*, etc.), along with anti-inflammatory/regulatory genes, predicted to be driven by monocyte-derived cells (macrophages and DCs) and T cells. ERB responses to MARV were also highly similar between individual bats (see **Supplementary Figure 1**) and to responses identified previously in ERB tissues and immune cells (except for the stronger responses seen in skin at the MARV inoculation site, which was expected given that the large viral bolus at this site presumably attracts a mass infiltration of immune cells) (6, 28). However, ERB responses showed two marked deviations from primate responses. First, the bat host frequently showed DEG induction and resolution occurring earlier than in primates. This likely reflects more effective control of virus replication and responses that regulate disease tolerance mechanisms (possibly via better-adapted viral sensing mechanisms that more quickly activate host genes) (6, 59–64), or perhaps that MARV immune evasion is more successful during early primate infection (47, 65–69). Either of these scenarios would give ERBs a critical advantage in modulating filovirus infection before it overwhelms the immune system. Indeed, administration of antiviral agents early in infection, particularly prior to symptom onset, often proves successful at counteracting filovirus replication and severe disease (70–77). Second, peak DEG FC in ERBs were at levels largely similar to, or substantially lower than, peak DEG FC in primates, depending on the specific study and gene, with a few exceptions of ERBs having stronger induction (e.g., *USP18*, *CCR5*). In certain cases, levels of regulation for genes like *C3*, *IFIT2*, *MX1* and *KLRK1* were 2- to 10-fold less intense in ERBs. While caution must be exercised when making specific quantitative interpretations between different systems and species, the overall qualitative trend appears internally consistent among these primate studies and agrees with prior data from MARV-infected ERBs that routinely shows exponentially lower viral loads in the blood and tissues compared to filovirus-infected primates, perhaps indicative of rheostat-like response gene activation in ERBs (6, 11, 21, 22, 38, 43, 52). Lower replication in ERBs suggests a role for effective host resistance mechanisms that actively limit MARV. Given that more pronounced ISG responses in primates are associated with severe disease outcomes (10, 17, 18, 38, 53, 78–80), an ability by ERBs to indirectly modulate DEG activation via better direct control of virus replication could help explain how the reservoir host prevents runaway immunopathology. Alternatively (or additionally), IFN antagonism by MARV may be better adapted to ERBs (26, 28, 47, 66), more precisely targeting specific IFN pathways and/or specific cell types and thus mitigating the risk of uncontrolled inflammation, which reflects the lock-and-key coevolution of specific viruses with reservoir but not spillover hosts. This notion is supported by a lack of ebolavirus replication in ERBs (22), likely not a reservoir for these viruses, which could conceivably be due to incompatible, ineffective antagonism. More research is needed to better understand the role of filoviral IFN antagonism in the context of natural host infection.

Several DEGs shared between ERBs and primates may be important specifically in the context of peripheral responses and/or involve pro- and anti-inflammatory genes and pathways indicative of both resistance and tolerance mechanisms, including some not previously identified in MARV-infected ERB tissues (6). DEGs include those with roles in: cytotoxic (CD8^+^) T cell/NK cell activation, proliferation and/or recruitment (inflammatory cytokine gene *IL15*, receptor gene *CCR5*, which *IL15* can signal through, *STAT1*/*2*, *TRAF2* and downregulated *FOS*, an AP-1 transcription factor component gene), T cell modulation or regulatory T cell (Treg) activation (*LGALS9* and immune checkpoint molecule gene *CD274*), NK cell activation (*IL15*, *TAP1* and downregulated *KLRK1*, an NK/T cell receptor gene that modulates their cytotoxicity), monocyte responses (*STAT1*/*2*, *ISG15*/*20*, *CCR5* and *MYC*), B cell maturation (*TAP1* and *TNFSF13B*) and apoptosis (pro-apoptotic *BID* and *TNFSF10* and anti-apoptotic *XAF1*), many of which represent blood responses typical during acute viral infections (10, 11, 38, 43, 52, 79, 81–89). This correlates cellularly with the proportional expansions of ERB NK cells, DCs and myeloid cells (monocytes/macrophages) estimated between 4-6DPI at peak viral load and gene induction via CIBERSORTx. The commonality of these DEGs regardless of host or filovirus indicates a functional conservation of both mammalian defense mechanisms, such as IFN signaling, T cell activation and apoptosis, and viral infection mechanisms, such as IFN antagonism, even when certain mechanisms may differ molecularly (e.g., antagonism by EBOV VP24 but by MARV VP40 (67, 90, 91)). For example, the combined regulation of *BID*, *TNFSF10* and *XAF1*, or the combined regulation of *CD274*, *FOS* and *KLRK1*, suggest conserved filovirus-induced apoptosis/necrosis, or modulation of T/NK cell activation/recruitment, respectively, while upregulation of *TNFSF13B*, and *IL15* with *CCR5*, suggest conserved host attempts to, respectively, stimulate B cell responses and activate T/NK cell recruitment to infection sites (e.g., liver) (11, 23, 53). T/NK cell recruitment may also be more effective in ERBs given their stronger *CCR5* induction. Some shared blood DEGs were previously identified in ERBs at the MARV inoculation site (e.g., *CCR5*, *LGALS9*, *CD274*, *BID*, *FOS*) *(* 6). Similarities between ERB blood and skin responses, including FC intensities, may reflect peripheral cells naturally responding to and infiltrating the inoculation site. Alternatively, they may reflect expression changes following virus and/or skin immune cells (or their signals) entering the blood that peripheral cells can then detect. Meanwhile, we found DEGs and related pathways shared between hosts that are directly or indirectly regulated by pro-inflammatory TNF (itself an activated pathway), including TNFR1 receptor superfamily members (*BID, BIRC2*, *CCR5*, *FOS*, *IRF7*, *MYC*, *PKR*, *TNFSF10* and *TRAF2*) *(* 92, 93) and TNFR2 receptor, TNFR2 non-canonical NFκB signaling, regulation of TNFR1, and IFNγ pathways. This shared regulation suggests that a TNF-mediated inflammatory response is not inherently an indicator of filovirus disease progression (18, 78, 83, 84, 94–96), but an important factor in natural mammalian antiviral responses. Indeed, the abundance of innate and adaptive pro-inflammatory DEGs and pathways identified even for ERBs is an important counterpoint against the prominent hypothesis within the field that viral control in these and other bat reservoirs is solely reliant on either anti-inflammatory tolerance mechanisms or constitutively-active IFN responses, rather than simply being due to an appropriate coordination of both pro- and anti-inflammatory responses (2, 59, 61, 62, 97–107). Further support includes enrichment of pro-inflammatory pathways like TLR, IL2/4, G-CSF/M-CSF and MHC-I antigen processing suggestive of peripheral T cell and monocyte activation and MHC-I antigen presentation (19, 20, 83, 86, 94, 108, 109). Normally, such T cells and anti-inflammatory Tregs are activated via the T cell receptor (TCR) and CD28 co-receptor. Activated helper (CD4^+^) and/or CD8^+^ T cells are the major inducers of immunoregulatory IL2, which in balance with IL15 promotes growth, development and survival of T, B and NK cells. Upregulated IL4, IL13 and TGFβ pathways are also anti-inflammatory; IL4 and IL13, which are produced by peripheral Th2 CD4^+^ T cells, promote antigen-specific T cell responses, amplify Th2 cell development, control pro-inflammatory cytokine production, regulate Th1/cytotoxic cell activities, and prevent tissue damage (82, 83, 85, 86, 89, 108–110).

Although the above pathways and constituent DEGs were shared between ERBs and disease hosts, many additional peripheral DEGs were uniquely activated or suppressed among primates but not perturbed in the bat reservoir, indicative of these pathways and other immunocellular activities being instead dysregulated in the spillover hosts. Several predominant trends emerge when analyzing these significant primate-specific DEGs. The consistency of response genes present in as many as all nine primate datasets (including *BATF*, *ICAM1*, *MKNK1*, *SOCS3*, *FAS*, *IL6*, *AKT3* and others) indicates a certain level of confidence in their predictability as putative markers of spillover host-specific filovirus disease, while filtering out common but less disease-relevant genes dysregulated across all infected mammalian hosts (e.g., *IFIT1*, *ISG15*, *OAS1*, *STAT1*). This suggests the potential ability of these primate-specific genes to act as a transcriptional signature across multiple primate hosts, one that would need to be independently confirmed in future studies. The most important overarching themes suggested by divergent primate peripheral responses to filoviruses can be defined by a lack of immunological control and a resistance/tolerance imbalance (3, 39, 62, 78, 94, 111, 112), either between pro- and anti-inflammatory responses or different immune cell types. Spillover host responses are often contradictory, with related genes being up- and downregulated simultaneously, and their mixed signals disrupt normal cellular functioning, protection from excessive inflammation, and efficient antiviral effector control of virus replication, all of which can contribute to pathogenesis and disease (11, 17, 18, 38, 96, 113). The most relevant peripheral responses dysregulated across primate studies were for ISGs and cytokines (including JAK/STAT, TLR, MAPK and TNF/NFκB signaling); T, B, NK, neutrophil and monocyte cells (including CD4^+^, CD8^+^, Th17 and regulatory T cells, macrophages and DCs); immune cell trafficking; and cell survival.

For ISG and cytokine responses, several primate-specific DEGs were pro-inflammatory, including *TNF*, *IL6*, *IL1B*, *MYD88*, *CCL2*/*3*, *IRAK2*, *TLR3/4*, *CXCL11*, *IL18* and *IL18R1*, some of which, such as *IL6*, *IL1B*, *CCL2*/*3* and *TNF*, are hallmarks of severe filoviral disease (*IL6* expression remained below background in ERB whole blood prior to and after MARV infection, though modest *IL6* expression has been previously measured by nCounter in naïve ERB tissues) (6, 38, 43, 81, 83, 94, 111, 114). The excessive inflammatory gene upregulation in filovirus-infected primates agrees with previous reports showing that they potentiate a cytokine storm that can lead to further immune cell dysregulation, tissue damage, hemorrhage, and eventually DIC and multiorgan system failure that often proves fatal (3, 83, 94, 113, 115). Indeed, the immune response studies used for our analysis involved infected NHPs that were moribund with similar inflammatory symptomology prior to reaching endpoint criteria (40, 43, 44, 51–54). Inflammation can be exacerbated by overactive IFN response signaling from the likes of *MX2*, *IFITM1*, *IFNGR1*/*2*, *IFNAR2*, *STAT3*, *IRF1*/*5* and *JAK2* genes, as well as NFκB pathway member genes *NFKB2*, *RELA* and *RELB*, for which TNF is a stimulator and the combination of which drives responses toward a Th1 inflammatory state (10, 18, 44, 83, 88, 94, 113). On the other hand, primate responses also show an increased number of dysregulated anti-inflammatory/regulatory DEGs not significantly changed in ERBs, including cytokine suppressor genes *SOCS1*/*3*, NFκB modulating genes *CEBPB*, *NFKBIA* and *TNFAIP3*, anti-inflammatory cytokine receptor genes *IL2RA*, *IL10RB*, *TGFBR2* and *IL4R*, RIG-I signaling repressor gene *DHX58* and TLR signaling repressor gene *IRAK3*. These responses may be indicative of negative feedback induced by the host to control inflammatory signaling but which are ultimately rendered ineffectual due to the overwhelming magnitude of inflammatory gene dysregulation, delayed timing in feedback gene expression or protein activities, functional suppression by filoviral proteins, or complex crosstalk with other immune genes or pathways that causes inadvertent propagation of inflammatory signals. Decoding the complex interplay between redundant, synergistic and/or antagonistic responses in filovirus-infected primates, including magnitude and kinetics, remains one of the field’s biggest challenges, with state-of-the-art techniques like single-cell sequencing and multi-omics holding promise in their future elucidation.

*IL6* is among the most prominent peripheral DEGs for primates, identified in all but one of the eight included studies, and shows one of the most dramatic changes in expression between fatal and non-fatal human cases, shifting from strong induction in fatal infections to a reservoir-like absence of induction in survivors (39). Such a trend was observed for only nine additional primate-specific DEGs: *BST2*, *MKNK1*, *CEBPB*, *IL18RAP*, *CSF1*, *CD163*, *NFKB2*, *CCL20* and *CEACAM1*. Of these, the less prevalent *CSF1*, *CD163*, *CEACAM1* and *CCL20* showed the biggest fold-change shifts between fatal and survivor cases, which may not indicate their relative importance over the other DEGs so much as reflect that human responses measured by Liu et al. sometimes had weaker intensities of gene induction than those measured in the studies of their NHP counterparts (possibly due to human patients having received far lower real-world inoculum dosages from differing routes) (39). However, previous reports have shown *IL6* and *CSF1* upregulation is linked to fatal human cases, while also showing that *CCL5*, which we found to be upregulated in ERBs but downregulated among primate studies, is associated with survival in pediatric cases (84, 95, 116, 117). Several other genes showed reduced, but not eliminated, up- or downregulation in human survivors, including *IFI35*, *SOCS1*/*3*, *IL18R1*, *CCL2*/*3*, *DHX58*, *CD28*, *VCAM1* and *CD40LG*. However, it is worth noting that many of these latter DEGs were not identified as significantly changed in some primate datasets and thus they may not be as intrinsically correlated with viral pathogenesis or disease progression, perhaps due to functional redundancy with other DEGs responsive in those infections. Nevertheless, these genes, and especially *IL6*, are worth further investigation as virulence determinants in which overall expression trends with disease outcome, particularly the analysis of molecular mechanisms governing their transcriptional regulation in ERBs.

Several primate-specific DEGs were related to cell survival, trafficking and neutrophil responses. Upregulated *FAS*, *CASP1*/*10*, *RIPK2*, *PYCARD* and *TNF*, as well as downregulated *AKT3*, *BCL2* and *CASP2*, play important roles in regulation of cell death and survival (11, 43, 83, 94, 113, 118–121). The imbalance in these processes could lead to excessive “bystander” apoptosis or necrosis, such as reported for T cells during primate infection (89, 118). Alternatively, these primate DEGs could send aberrant survival signals to infected monocytes and DCs that allow for more of them to successfully invade tissues, where they may seed inflammatory lesions that exacerbate pathology. Indeed, multiple primate-specific DEGs are also involved in immune cell trafficking to lymphatics or tissue sites of infection, such as upregulated adhesion molecule genes *ICAM1*/*VCAM1*, chemokine genes *CCL2*/*3*/*8*/*20* and *CXCL2*/*11*, scavenger receptor gene *CD163*, and *ITGAM* (also called *CD11b*, a classical DC marker gene), and downregulated TCR co-receptor gene *CD28*, Th2 chemokine receptor gene *CCR3* and T cell activation marker gene *CD40LG (* 16, 83, 89, 110, 113). These upregulated DEGs are known to promote recruitment of immune cells to infected tissues, possibly enhancing the extent of the monocyte/DC infiltration as suggested above along with inflammatory responses initiated by other recruited cell types (11, 19, 20, 85, 87, 119). Conversely, these downregulated DEGs likely affect which cell types migrate, suppressing the activation and recruitment of B cells and T cells, including anti-inflammatory Th2 cells, which augments the Th1-skewed state described earlier. Meanwhile, several DEGs affect neutrophil responses, including *CXCL2*/*11*, *ITGAM*, *FAS*, *AKT3*, *NFKBIA*, fibrinolysis genes *PLAU*/*PLAUR* and kinase gene *MAPK14*. Their dysregulation could lead to overabundant neutrophil tissue infiltration, phagocytosis, turnover/survival, and tissue damage via neutrophil-induced reactive oxygen species (44, 81, 112, 122–124). In the blood of MARV-infected ERBs, we saw only a modest and transient decrease in neutrophil proportion at peak infection based on CIBERSORTx analysis, indicative of a well-regulated transmigration of a more appropriate number of neutrophils to tissues.

We identified numerous primate-specific DEGs involved in monocyte and B cell responses. Monocyte-derived cells (macrophages and DCs) are among the most critical immunocellular responses dictating both filovirus control and inflammatory response regulation, due to their dual roles in pathogenesis as early peripheral immune cell responders and as early virus infection targets (11, 19, 20, 85, 87, 96, 119, 121). Dysregulation related to monocyte, macrophage and DC activation/signaling include DEGs for: cytokines (*IL1B*, *IL6* and *IL18*), signaling (*MYD88*, *TLR3*/*4*/*8* and *RELA*/*B*), recruitment (particularly monocyte chemoattract protein genes *CCL2*/*8* and macrophage inflammatory protein genes *CCL3*/*20*), cell markers (*CD14*, *ITGAM* and *CD163*), cell stimulation (*CSF1*, *CSF2RB*, *CEBPB* and downregulated *CSF1R*, which may offset *CSF1*-mediated activation and prevent proper development) and cytotoxicity or anti-inflammation (IgG receptor genes *FCGR1A*/*2A*, *ITGAM*, downregulated Th2-dependent lipoxygenase *ALOX15*, and cell death genes *FAS*, *RIPK2* and *CASP*s, in which overabundant apoptosis triggers phagocytic activity) (19, 83, 85, 87, 94, 96, 114, 119). Overall, these DEGs in primates suggest enhanced monocyte activation, differentiation again skewed toward a pro-inflammatory phenotype, and likely robust expansion as first-line responders and co-opted filoviral targets. Infection in ERBs conversely showed moderate, transient expansion of myeloid cells (monocytes/macrophages) and DCs at peak infection via CIBERSORTx. Previous transcriptional data from ERB bone marrow-derived DCs has shown a similar response profile to the blood, suggesting that these cells are not dysregulated in the bat reservoir (28). Peripheral B cell responses are also perturbed in primates, including those affecting activation and proliferation (*BTK*, *SYK*, *PRKCB*/*D*, and downregulated marker genes *CD40LG* and *CD19*), maturation and differentiation (*NOTCH1*), antibody production (*IL10RB*), migration (*CXCL11* and *CCL20*) and survival (*FAS*, *TNFAIP3* and *TNFRSF17*) *(* 16, 52, 79, 96, 125–128). The conflicting array of response genes for B cells indicates an attempt in primates to induce robust humoral responses for plasma cell development and antibody production, but which filoviruses suppress through stimulation of cell death mechanisms and repressive signaling. In the MARV bat host, B cell responses appear to be less critical to overall virus control than other cell types, including antibody responses, which are non-neutralizing and may be secondary to rapid innate responses (6, 23, 33, 34, 129). It will be of interest in future studies to investigate the antiviral roles of monocytes and B cells in MARV-infected ERBs, which remain unclear.

Above all other cell types, T cells and NK cells appear to be particularly targeted by primate-specific peripheral immune responses to filoviruses. This is evidenced by upregulated DEGs with roles in: T cell activation, proliferation and function, either generally (*ICAM1*/*VCAM1* and *PRKCB*/*D*) or specifically for Th1 cells (*STAT1*/*3*, *JAK2*, *TBK1*, *TLR3*/*4* and *IL2RA*), Th2 cells (*IL4R*), Tregs (*IL2RA*, *SOCS1* and *IL10RB*) or Th17 cells (*IL6* and *STAT3*); migration (*CCL2*/*3*); regulation and exhaustion (*HAVCR2*); antigen presentation (*TAP2* [CD8^+^ cells]); and NK cell activation and function (*IL2RA*, *IL18R1*/*RAP*, *TNF*, *HAVCR2*, *IFNGR1*/*2*, *IFNAR2* and indirectly increased cytotoxic activity via *FAS*) *(* 39, 40, 78, 79, 82–86, 92, 94, 108, 113). Downregulated DEGs involved in these responses were also abundant in primates: receptor signaling and activation (*CD28*, *CD247*, *CD3D*/*E*, *CD8B*, *CD40LG*, *DPP4*, *ZAP70*, *LCK* and *FYN*), recruitment (*CCL5* and *CCR3*), antigen presentation and cell priming (downregulated *HLA-DMA*/*DOB* [CD4^+^ cells]), and NK cell function (*KLRB1*/*D1*/*G1*) (16, 39, 79, 82–86, 92, 94, 110, 113, 130, 131). In total, DEGs involved in T/NK cell responses represent well over a third of the 119 primate-specific DEGs shared by at least four datasets. These responses suggest that, in primates, T cells are mainly downregulated as a whole (declines in common T cell markers such as TCR genes *CD3D*/*E* and *CD247* [*CD3Z*], *CD8*, *CD40LG* and costimulatory molecule gene *CD28*), which along with dysregulation of several cell death/apoptosis genes such as *FAS* and *CASP*s, correlates with the bystander lymphocyte apoptosis seen during primate infection (10, 11, 16, 86, 89, 118), likely a filovirus-mediated mechanism meant to limit T cell antiviral responses. Some of the upregulated DEGs suggest that any peripheral T cells not targeted for apoptosis are ostensibly driven, much like the ISG/cytokine, trafficking and myeloid cell responses, toward pro-inflammatory Th1 and Th17 responses that may also promote cytotoxicity (despite the downregulation of *CD8* in some datasets) (78, 79, 82, 83, 88). This could fuel a feedback loop of T cells directly or indirectly contributing to their own apoptosis. Meanwhile, efforts presumably by the host to activate anti-inflammatory Th2 and Treg cells likely are not enough to maintain control over these pro-inflammatory and cell death signals. This is further supported by the DEGs related to NK cell responses, which largely favor their stimulation and promote their cytotoxicity (with the three downregulated regulatory KLR genes no longer able to inhibit NK cell and in some cases T cell function), contributing to tissue inflammation and apoptosis/necrosis (10, 130, 132). On the other hand, in the MARV bat reservoir, peripheral T cell responses do not appear to be dysregulated, and there is little evidence of bystander apoptosis (6, 22, 23, 58). Indeed, CIBERSORTx analysis shows that T cell proportion in the blood remains constant pre- and post-MARV infection, along with an appreciable but transient proportional NK cell expansion that returns to baseline by 7DPI, right after blood viral loads peak. We speculate that T cell responses are an essential part of a nevertheless critical pro-inflammatory antiviral response to MARV in ERBs, one that, unlike in filovirus-infected primates, is cell-specific and highly controlled, likely involves monocytes, and directly limits viral burden, which quickly leads to clearance.

Taken together, the transcript-level observations between filovirus reservoir and spillover hosts reported herein help to distinguish the specific immune responses incurred during respective infection by a coevolved or foreign agent. While these hosts share many critical genes to control filovirus infection, only the bat reservoir has adapted coordinated innate and adaptive responses capable of a fine-tuned balance between direct antiviral pro-inflammatory resistance and indirect anti-inflammatory regulation of this resistance, actively limiting viral replication and disease while maintaining control over the expression of various ISG, cytokine, monocyte, T cell and NK cell genes. However, primates infected by non-adapted filoviruses inadvertently activate or suppress too many of these immune-related genes, leading to a dysregulated and overactive response, leading to a dysregulated and overactive response characterized by hijacked monocyte responses, impaired normal T and NK cell antiviral activities, and an immune system skewed toward an amplified pro-inflammatory and cytotoxic environment that is known to be responsible for life-threatening tissue damage, coagulopathies and organ failure (3, 78, 89, 94, 112, 113). This study highlights the exquisitely refined immune responses of a filovirus natural host, while also providing a comprehensive evaluation of the specific peripheral immune-related genes, and the cell types, processes and pathways those genes regulate, that are uniquely dysregulated in primate spillover hosts and the most likely to contribute to severe filoviral disease. Going forward, it will be vital to ascertain the exact molecular mechanisms dictating regulation of transcriptional activation or repression of these genes in both filovirus hosts, particularly early in infection, as well as to identify any gene product functions or targets that influence the progression of their deviating immune responses. Such lessons could spur development of novel anti-filoviral interventions capable of modulating primate responses to be more reservoir-like, better controlling viral replication and/or mitigating pathological inflammation, as well as help to discern any conserved immunological trends that are applicable to the virulence-determining response dynamics between other zoonotic reservoir and spillover hosts.

## Data availability statement

The original contributions presented in the study are included in the article/**Supplementary Materials**. Further inquiries can be directed to the corresponding authors.

## Ethics statement

The animal study was approved by CDC Institutional Animal Care & Use Committee (IACUC), in conjunction with oversight and assistance from the Animal Care and Use Program Office (ACUPO) and Comparative Medicine Branch (CMB). The study was conducted in accordance with the local legislation and institutional requirements. Ethical approval was not required for the study involving humans in accordance with the local legislation and institutional requirements. Written informed consent to participate in this study was not required from the participants or the participants' legal guardians/next of kin in accordance with the national legislation and the institutional requirements.

## Author contributions

JG: Conceptualization, Formal analysis, Investigation, Writing – original draft, Writing – review & editing. CA: Formal analysis, Writing – review & editing. AS: Investigation, Writing – review & editing. BA: Investigation, Writing – review & editing. TS: Investigation, Writing – review & editing. JS: Investigation, Writing – review & editing. JH: Investigation, Writing – review & editing. JC-M: Investigation, Writing – review & editing. MS-L: Funding acquisition, Writing – review & editing. GP: Funding acquisition, Writing – review & editing. JT: Conceptualization, Funding acquisition, Investigation, Writing – review & editing. JP: Conceptualization, Formal analysis, Investigation, Writing – review & editing.

## Supplementary material

Supplementary Figure 1 Differential expression of select DEGs in whole blood of individual MARV-infected ERBs. Normalized ERB immune gene counts (see **Supplementary Data File 1**) were used to assess consistency of linear FC for representative canonical DEGs across the five study bats (each bat denoted by indicated color) and correlate response intensities to individual levels of MARV replication (see **Figure 1**). FC was obtained by dividing counts of each gene for each bat at indicated time points post-infection by the averaged baseline count of that gene for these five bats prior to infection.
