# Supplementary figures and images for: Peripheral immune responses to filoviruses in a reservoir versus spillover hosts reveal transcriptional correlates of disease
Source: Front Immunol. 2024 Jan 8;14:1306501. doi: 10.3389/fimmu.2023.1306501 (PMC10800976; doi:10.3389/fimmu.2023.1306501)

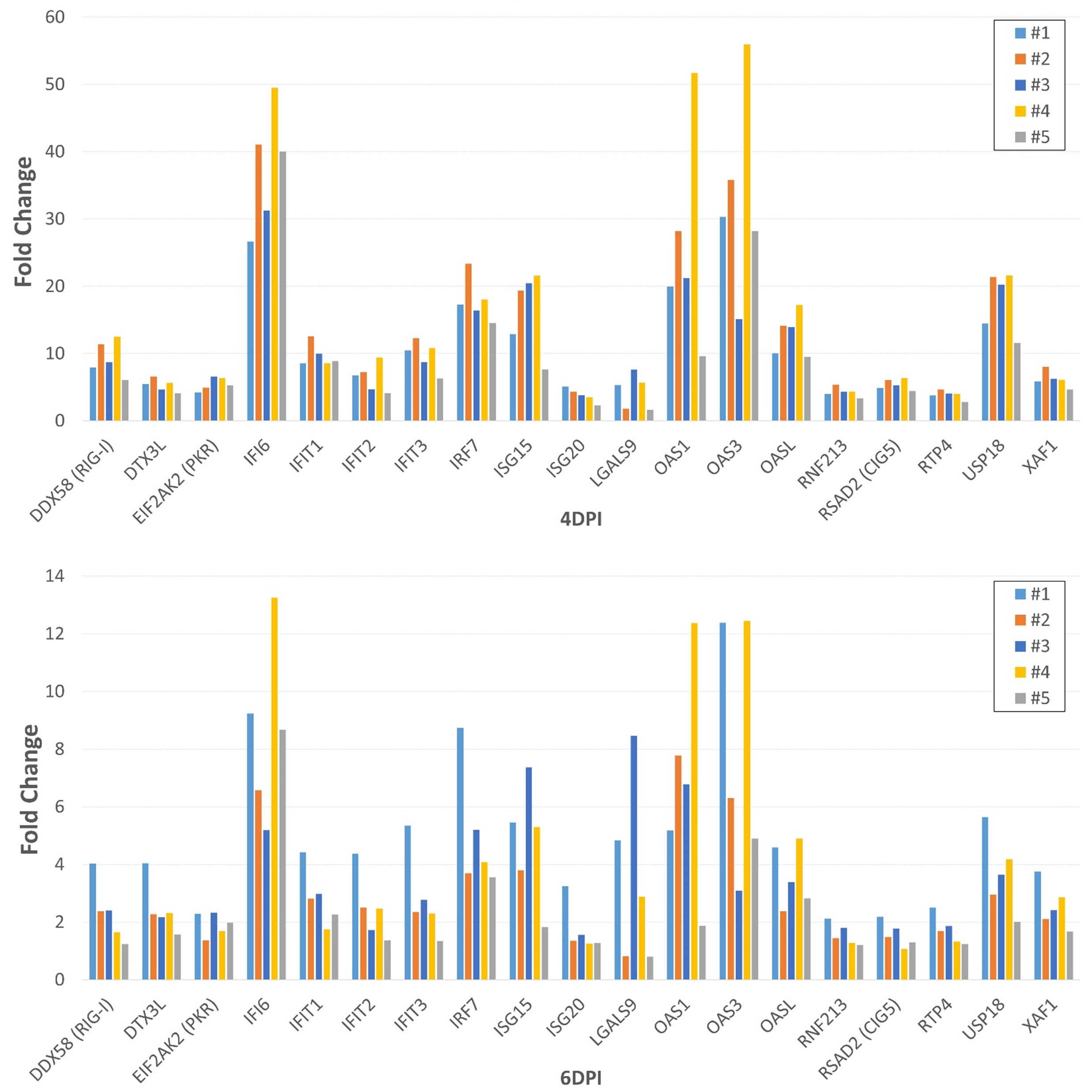

Supplement: Supplementary Figure 1 — Differential expression of select DEGs in whole blood of individual MARV-infected ERBs. Normalized ERB immune gene counts (see Supplementary Data File 1 ) were used to assess consistency of linear FC for representative canonical DEGs across the five study bats (each bat denoted by indicated color) and correlate response intensities to individual levels of MARV replication (see Figure 1 ). FC was obtained by dividing counts of each gene for each bat at indicated time points post-infection by the averaged baseline count of that gene for these five bats prior to infection. [file Image_1.jpeg]
